# Using the Beck Depression Inventory to Assess Anhedonia: A Scale Validation Study

**DOI:** 10.1177/10731911231164628

**Published:** 2023-04-11

**Authors:** Ashby B. Cogan, Jacqueline B. Persons, Ann M. Kring

**Affiliations:** 1 University of California, Berkeley, USA; 2 Oakland Cognitive Behavior Therapy Center, CA, USA

**Keywords:** anhedonia, Beck Depression Inventory (BDI), scale validation, internal consistency, convergent validity, discriminant validity, measurement-based care

## Abstract

Anhedonia is central to several psychological disorders and a frequent target of psychosocial and pharmacological treatments. We evaluated the psychometric properties of two widely used anhedonia measures derived from the Beck Depression Inventory: a 3-item (BDI-Anh3) and a 4-item version (BDI-Anh4). We evaluated these measures in a large undergraduate sample, a community sample, and a clinical sample. Both the BDI-Anh3 and the BDI-Anh4 showed adequate internal consistency, with BDI-Anh4 performing somewhat better, across the three samples. Both measures showed good convergent and discriminant validity, even after controlling for shared variance with other items on the BDI. These findings indicate that both measures have sufficient reliability and validity to support their use by researchers and clinicians.

Anhedonia, a deficit in the experience of pleasure, is a central feature of many psychological disorders including depression, schizophrenia, posttraumatic stress disorder, substance use disorders, and eating disorders (American Psychiatric Association, 2022; Dolan et al., 2022; Garfield et al., 2014; Kring & Barch, 2014; Kring & Elis, 2013; Olin et al., 2022). It is a predictor of poor outcome of pharmacotherapeutic treatment for depression (e.g., Khazanov et al., 2020; McMakin et al., 2012; Uher et al., 2012) and of psychosocial treatment for cocaine dependence (Crits-Christoph et al., 2018), depression (Khazanov et al., 2021), and opioid use disorder (Kiluk et al., 2019). Anhedonia is also an important individual difference in non-clinical populations (e.g., Gard et al., 2006; Gooding & Pflum, 2014).

Anhedonia has been heterogeneously conceptualized in the literature. For example, some conceptualize anhedonia in terms of its source—for example, physical, social, and other categories of pleasure, such as intellectual pleasure (e.g., Berenbaum, 2002; Chapman et al., 1976). Other conceptualizations are influenced by neuroscience (e.g., Berridge & Kringelbach, 2011) and focus on the time course of pleasure, including anticipatory and consummatory components of sensory (e.g., Gard et al., 2007; Kring & Barch, 2014) and social (e.g., Rademacher et al., 2010) pleasure.

Given anhedonia’s importance, particularly in clinical contexts, a brief and psychometrically sound measure of anhedonia could be useful as a support to the data-driven mental health provider in selecting a course of treatment, staying abreast of symptom changes, and course-correcting to avoid treatment failure (Lewis et al., 2018; Persons, 2008). Although several measures of anhedonia have been developed, among them the Temporal Experience of Pleasure Scale (TEPS; Gard et al., 2006) and the Snaith Hamilton Pleasure Scale (SHAPS; Snaith et al., 1995), they are not often used in clinical contexts for either practical reasons (e.g., length) or because there is limited evidence supporting their use in clinical contexts. A brief, psychometrically sound scale to assess anhedonia has the potential to be helpful to clinicians.

The Beck Depression Inventory (BDI), first released over 60 years ago (Beck, 1961; Beck et al., 1979) and revised most recently in 1996 (BDI-II; Beck, Steer, & Brown, 1996), is commonly used in clinical settings and includes items that assess anhedonia. Joiner and colleagues (2003) proposed assessing anhedonia with three items from the BDI: Items 4 (loss of satisfaction/enjoyment), 12 (loss of interest), and 21 (loss of interest in sex). They reported an alpha coefficient for the three items of .57. A 4-item version from the BDI-II was proposed by Pizzagalli et al. (2005), who added Item 15 (effort/energy) to the 3-item measure and reported an alpha of .60 for the 4-item measure. Since these measures were proposed, nearly 20 studies have used the 3-item measure (Ballard et al., 2017; Carbajal et al., 2017; Crits-Christoph et al., 2018; Fan et al., 2021; Hasler et al., 2010; Kashdan et al., 2006, 2007; Leventhal et al., 2006; Loas et al., 2016, 2018, 2019; Martínez-Vispo et al., 2020; Nizet et al., 2018; Peechatka et al., 2015; Pushkarskaya et al., 2019; Santopetro, Brush, Bruchnak, et al., 2021; Santopetro, Brush, Burani, et al., 2021; Steer, 2011; Yan et al., 2011), and at least 16 others have used the 4-item measure (Barr et al., 2008; Blain et al., 2021; Bogdan & Pizzagalli, 2006; Carl et al., 2016; Crowther et al., 2015; Lopez-Gamundi & Wardle, 2018; Pizzagalli et al., 2005, 2008, 2009; Solibieda et al., 2021; Treadway et al., 2009; Walsh et al., 2017, 2019; Winer et al., 2014; Yang et al., 2020).^1,2^

Although the 3-item and 4-item BDI-derived anhedonia scales have been frequently used by researchers and have the potential to be useful to clinicians, very limited data about the psychometric properties of the scales are available. Although some studies using these scales report their reliability (e.g., Joiner et al., 2003; Kashdan et al., 2006; Pizzagalli et al., 2005), most do not. Although scale validation was not a study aim, a few studies have reported correlations between the BDI-derived anhedonia measures and other anhedonia scales, providing some convergent validity support. Leventhal et al. (2006) reported that the 3-item scale was modestly related to other measures of pleasure, including the SHAPS, the Chapman Physical Anhedonia scale (Chapman et al., 1976), and the Fawcett-Clark Pleasure scale (Fawcett et al., 1983) in a large sample of undergraduates (*r* = −.33, .14, and −.28, respectively). Similarly, Treadway et al. (2009) found the 4-item version to be moderately correlated with the SHAPS (−.38) and the Chapman anhedonia scales, and modestly correlated with the positive affect scale (−.28) of the Positive and Negative Affect Schedule (PANAS; Watson & Clark, 1988) in an undergraduate sample. In a clinical sample of people with mood or anxiety disorders, Loas et al. (2016) reported modest correlations between the 3-item scale and the TEPS anticipatory (−.20) and consummatory (−.27) scales. Notably, these three studies also reported strong correlations between the BDI-derived anhedonia measures and the remaining BDI items (*r* values ranging from .72–.82). Two of these studies reported discriminant validity correlations, that is, correlations with measures not expected to be related to anhedonia. Treadway et al. (2009) reported a non-significant correlation (.21) between the 4-item anhedonia scale and the negative affect scale of the PANAS, and Leventhal et al. (2006) reported a moderate correlation (.39) between the 3-item version and the Beck Anxiety Inventory (Beck et al., 1988). In short, a systematic evaluation of the BDI-derived scales’ psychometric properties and validity has not yet been conducted, and doing so was the primary motivation for the current study.

We sought to systematically evaluate the psychometric properties of both the 3- and 4-item BDI-derived anhedonia scales, herein referred to as BDI-Anh3 and BDI-Anh4. We used a derivation and replication design to test the generalizability of our findings. Specifically, we included a large derivation sample (undergraduates) and two replication samples: (a) community residents recruited via Amazon Mechanical Turk (AMT), and (b) a clinical sample receiving outpatient treatment at a center for cognitive therapy to assess the internal consistency and convergent and discriminant validity of both scale versions.

We hypothesized that both BDI-Anh3 and BDI-Anh4 would demonstrate convergent validity, as indicated by correlations with other anhedonia measures and positive affect. In addition, we hypothesized that both scales would demonstrate discriminant validity, as indicated by correlations with constructs not related to pleasure or anhedonia, including self-esteem, anxiety, stress, and negative affect. Furthermore, we hypothesized that both scales would demonstrate convergent and discriminant validity even after controlling for shared variance with other items on the BDI, a stronger test of both convergent and discriminant validity.

## Method

The study was approved by the Ethics Committee of the University of California, Berkeley (the undergraduate and community samples), and the Behavioral Health Research Collaborative, which reviewed the procedures used to establish and maintain the Naturalistic CBT Archival Database (the clinical sample).

### Participants

**Figure 1.**
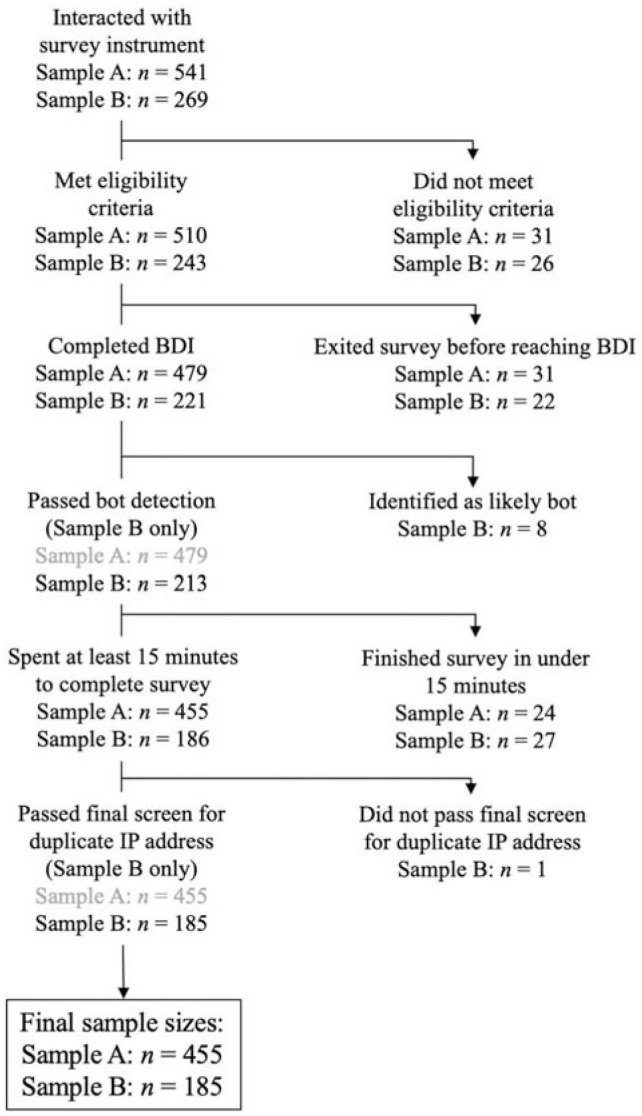
Diagram Showing Inclusion, Samples A and B.

Sample A, the *derivation* sample, consisted of undergraduate students (*n* = 455) recruited from a large West Coast university. Sample B, a community *replication* sample, consisted of community members (*n* = 185) recruited through AMT (Figure 1). Sample C, a clinical *replication* sample (*n* = 1,043), consisted of adults receiving psychotherapy primarily for mood and anxiety disorders at a cognitive behavior therapy (CBT) center. Data collection for Samples A and B took place online. Data from Sample C were collected during the course of participants’ psychotherapy. Eligibility criteria were English fluency, age over 18, and U.S. residency.

Because we wanted to ensure that participants had carefully attended to the task and followed instructions, we used conservative inclusion criteria. For Sample A, 510 participants met our initial eligibility requirements. Participants were excluded if they did not complete the BDI (*n* = 31) or completed the entire set of questionnaires in less than 15 min (*n* = 24), which we deemed to be insufficient time to complete all the included measures. Thus, the final *n* for Sample A consisted of 455 undergraduates. Participants received course credit for participation.

Sample B was recruited via AMT. Because our other two samples consisted of U.S. residents, we used AMT settings to restrict the study’s visibility to participants who used a United States IP address. Furthermore, we restricted access to those who had established their reputations on AMT by completing at least 50 tasks with a 95% acceptance rate, consistent with AMT sampling guidance (e.g., Peer et al., 2014). Of the 243 initially eligible participants, 22 did not complete the BDI; eight more were excluded by a suspicious-ISP detection algorithm (Prims et al., 2018). Finally, we excluded 27 people who completed the set of questionnaires in less than 15 minutes and one additional person with an IP address that was a duplicate of another, suggesting it was not the work of a unique participant. The final *n* for Sample B was thus 185. Participants received $5.00 for their participation in the approximately 30-min study, consistent with the AMT community norm for academic hosts (“requesters”) to pay at least $.10 per minute of work (Guidelines for Academic Requesters, 2014).

Participants in Sample C gave written consent to provide data for research during cognitive-behavioral therapy they received at a private cognitive behavior therapy center. We studied baseline (i.e., first therapy session) from *n* = 1,043 patients.

Available demographic information for all samples is presented in Table 1. We followed updated guidelines on reporting race and ethnicity, using “multiracial” for participants who identified as more than one race (Flanagin et al., 2021).

**Table 1:** Demographic Characteristics for All Samples.

	Sample A (*n* = 455)		Sample B (*n* = 185)		Sample C (*n* = 1,043)	
Characteristic		*n*	%	*n*	%	
Gender (female)	339	75	105	57	626	60
Ethnicity (Hispanic)	56	12	7	4	30	3
Race						
American Indian or Alaska Native	2	<1	3	2		
Asian	282	62	6	3	55	5
Black	3	1	11		21	2
Native Hawaiian or Pacific Islander	2	<1	1	6		
White	95	21	158	85	830	80
Multiracial/Other	54	12	5	3	32	3
Not provided	17	4	1	<1	75	7
Education						
Less than high school	2	<1			9	1
High school/GED	73	16	26	14	62	7
Some college	306	67	50	27	160	57
Bachelor’s degree	21	5	44	24	354	34
Advanced degree	2	< 1	15	8	336	32
Not provided	51	11	50	27	122	12
Marital status						
Married	6	1	80	43	315	30
Never married/single	447	98	78	42	508	49
Divorced	1	.2	22	12	70	7
Widowed			5	3	12	1
Other					88	8
Not provided	1	.2			30	3

*Note.* Advanced degree includes master’s degree or higher. In Sample C, Other Marital Status refers to either living with a partner or currently separated.

### Measures and Procedures

Participants in the clinical replication sample completed a paper version of the BDI. Participants in the derivation and community replication samples completed the BDI and additional measures to assess convergent and discriminant validity, presented in the same order to all participants, online via the survey platform Qualtrics. Participants in the derivation and community replication samples completed the BDI without Item 9, which assesses suicidality.^3^ Although participants in the clinical replication sample completed the full 21-item BDI, we removed Item 9 from our analyses.

#### Convergent Validity Measures

We included measures of anhedonia and positive affect to assess the BDI-derived anhedonia measures’ convergent validity. The measures we included each assess distinct facets of the capacity to experience pleasure (sensory, social, general), and none has exact overlap with all the facets seen in the BDI-derived anhedonia scales; thus, we could not make clear predictions about what measure might show the strongest test of convergent validity. We predicted that BDI-Anh3 and BDI-Anh4 would be positively correlated with each of the measures of anhedonia and negatively correlated with measures of positive affect. To test the first hypothesis, we assessed anticipatory and consummatory physical pleasure using the Temporal Experience of Pleasure Scale (TEPS; Gard et al., 2006). The TEPS is an 18-item measure that queries about physical or sensory pleasure. There are 10 anticipatory pleasure items (TEPS-Ant) and eight consummatory pleasure items (TEPS-Con). We assessed general pleasure using the Snaith-Hamilton Pleasure Scale (SHAPS; Snaith et al., 1995), which includes 14 items that address physical, sensory, and social pleasure. We also assessed reductions in social pleasure using the brief version of the Social Anhedonia Scale (SAS; Reise et al., 2011), which includes 24 true-false items that assess diminished pleasure in the social domain. To test the hypothesis that measures of anhedonia would be negatively correlated with measures of positive affect, we assessed positive affect (PA) using the PANAS (Watson & Clark, 1988). The PA scale comprises 10 items measuring the extent to which the person has experienced high arousal positive emotions. Samples A and B completed all measures; a small subset of Sample C completed the PANAS.

#### Discriminant Validity Measures

We included measures of constructs putatively unrelated to anhedonia to assess the BDI-derived anhedonia measures’ discriminant validity. We assessed self-esteem with the Rosenberg Self-Esteem Scale (RSES; Rosenberg, 1965), which is made up of 10 items measuring trait self-worth. We assessed anxiety using the 21-item Beck Anxiety Inventory (BAI; Beck et al., 1988), which measures cognitive, affective, and somatic symptoms of anxiety. We further assessed anxiety and stress using the Anxiety and Stress subscales of the 21-item Depression Anxiety and Stress Scales (DASS; Lovibond & Lovibond, 1995); the DASS-Anxiety subscale has seven items, and the DASS-Stress subscale has seven items. Finally, we assessed negative affect (NA) using the 10-item NA scale from the PANAS; this scale asks participants to report the extent to which they have experienced high arousal negative emotions over the past week. Samples A and B completed all measures; a small subset of Sample C completed the PANAS, and an even smaller subset completed the BAI.

### Data Analytic Strategy

Data analyses were conducted using SPSS, version 27 (IBM Corp., 2020). We assessed internal consistency in all three samples using Cronbach’s alpha, α, and McDonald’s omega, ω (Cronbach, 1951; McDonald, 1999). We assessed the convergent and discriminant validity of the BDI-derived scales in two ways. The first was through a simple correlation: did the BDI-derived measures correlate with other measures of pleasure and positive affect, and did they fail to correlate with putatively distinct constructs? The second was through partial correlations. Because of the high degree of correlation among *all* the BDI items, we conducted partial correlations to determine whether there were any unique relationships between the anhedonia items and the measures we used to assess convergent and discriminant validity (i.e., whether there were relationships not otherwise accounted for by the entire BDI).

Thus, for both BDI-Anh3 and BDI-Anh4, we assessed convergent and discriminant validity with zero-order Pearson’s correlations as well as with partial correlations that controlled for shared variance with the remaining BDI items. Most convergent and discriminant validity analyses were conducted with the derivation and community replication samples. We used the guidelines for interpreting the magnitude of correlational effect sizes outlined by Cohen (1988, 1992): correlation and partial correlation coefficients <.30 were considered modest, .30–.49 were considered moderate, and >.50, strong. We set the significance levels on all tests to *p* < .01 (two-tailed) for a conservative approach to interpretation and to indicate support for convergent and discriminant validity. Reported correlations remained significant at *p* < .01 after applying corrections for multiple comparisons (whether Bonferroni or the Benjamini–Hochberg false discovery rate procedure).

## Results

### Group Differences, Internal Consistency, and Scale Correlations

Descriptive statistics for the BDI-derived measures are provided in Table 2. Not surprisingly, participants in the clinical sample (Sample C) scored higher on both BDI-derived anhedonia scales and the BDI total score (all *p* < .001) compared with undergraduates (Sample A) and community participants (Sample B). We observed one statistically significant difference between Samples A and B: Those in the AMT community sample (B) scored higher on BDI-Anh3 than did those in the undergraduate sample (A), *t*(635) = 2.69, *p* = .007. We found no statistically significant gender differences in either the 3-item or 4-item scale in any of the samples. In addition, neither scale was statistically significantly correlated with marital status or years of education in any of the samples. Internal consistency of BDI-Anh3 and BDI-Anh4 was adequate across all samples. Alphas for the 4-item measure were .66, .74, and .71 for Samples A, B, and C, respectively, and thus were mostly above .70, which is generally considered acceptable internal consistency (Cicchetti, 1994).

**Table 2:** Descriptive Statistics for the BDI, BDI-Anh3, and BDI-Anh4 Across Samples.

	Sample A (*n* = 455)			Sample B (*n* = 185)			Sample C (*n* = 1043)		
Measures	*M* (*SD*)	α	ω	*M* (*SD*)	α	ω	*M* (*SD*)	α	ω
BDI total	9.81 (8.55)	.91	.91	9.77 (10.11)	.94	.94	16.08 (9.68)	.89	.89
BDI-Anh3	1.24 (1.41)	.55	.61	1.60 (1.85)	.68	.69	2.57 (2.00)	.65	.65
BDI-Anh4	1.89 (1.91)	.66	.69	2.05 (2.32)	.74	.75	3.64 (2.55)	.71	.71

*Note.* BDI = Beck Depression Inventory; BDI-Anh3 = 3-item BDI anhedonia scale (Items 4, 12, 21); BDI-Anh4 = 4-item BDI anhedonia scale (Items 4, 12, 15, 21). BDI Total does *not* include Item 9, suicidality, for any sample. *SD* = standard deviation; α = Cronbach’s alpha; ω = McDonald’s omega.

We computed correlations between the BDI-derived anhedonia scales and the remaining BDI items (see Table 3). Two things are noteworthy about these relationships. First, the anhedonia items are strongly correlated with the other BDI items. The magnitude of these correlations supports our decision to conduct partial correlations between the BDI-Anh scales with the convergent and discriminant validity measures to provide a more stringent test of the BDI-Anh scales’ validity than other investigations have previously offered. Second, BDI-Anh4 was significantly more strongly related to the remaining 16 BDI items than was BDI-Anh3 to the remaining 17 BDI items for Samples A (*z* = 3.42; *p* = .0006) and C (*z* = 2.94, *p* = .003). For Sample B, this difference approached significance (*z* = 1.83, *p* = .067).

**Table 3:** Correlations Between BDI-Anh Scales and Remaining BDI Items.

Measures	Sample A	Sample B	Sample C
BDI-Anh3 & remaining 17 items	.66	.76	.64
BDI-Anh4 & remaining 16 BDI items	.77	.83	.71

*Note.* BDI = Beck Depression Inventory; BDI-Anh3 = 3-item BDI anhedonia scale; BDI-Anh4 = 4-item BDI anhedonia scale.

All correlations significant at *p* < .00001.

Finally, we computed inter-item correlations for items in BDI-Anh3 and BDI-Anh4 for each sample. For BDI-Anh3, the mean inter-item correlations were .28, .43, and .39 for Samples A, B, and C, respectively. For BDI-Anh4, mean inter-item correlations were .31, .43, and .39 for the three samples.

### Convergent Validity

To test our hypothesis that both BDI-Anh3 and BDI-Anh4 would demonstrate convergent validity, we computed correlations and partial correlations between the BDI-derived anhedonia scales and other measures of anhedonia and positive affect, as shown in Table 4. When zero-order correlations were examined, in general, both BDI-Anh3 and BDI-Anh4 were moderately or modestly related to anticipatory and consummatory anhedonia as assessed by the TEPS, social anhedonia (SAS), general pleasure (SHAPS), and positive affect (PA) for both the derivation and community replication samples. The signs of all correlations were in the expected direction; for example, the BDI-derived anhedonia scales were negatively correlated with scores on the Positive Affect scale of the PANAS. In addition, these correlations were comparable across both BDI-derived measures. Correlations between the BDI-derived measures and consummatory pleasure assessed by the TEPS for Sample B were significant at the *p* = .05 level of significance, but not at the more conservative significance level we adopted for the study.

**Table 4:** Convergent Validity of BDI-Anh3 and BDI-Anh4.

	Zero-order correlations				Partial correlations			
	BDI-Anh3		BDI-Anh4		BDI-Anh3		BDI-Anh4	
Measures	A	B	A	B	A	B	A	B
TEPS-Ant	−.31*	−.20*	−.29*	−.23*	−.24*	−.05	−.23*	−.09
TEPS-Con	−.25*	−.17	−.25*	−.19	−.18*	−.03	−.19*	−.03
SHAPS	.25*	.43*	.26*	.44*	.10	.12	.07	.08
SAS	.44*	.41*	.41*	.40*	.33*	.21*	.30*	.18
PA	−.37*	−.28*	−.42*	−.34*	−.16*	.01	−.21*	−.07

*Note.* Partial correlations are between each BDI-derived anhedonia scale and other measures, controlling for the other items on the BDI. BDI-Anh3 = 3-item BDI anhedonia scale; BDI-Anh4 = 4-item BDI anhedonia scale; A = Sample A (*n* = 455), the derivation sample of undergraduates; B = Sample B (*n* = 185), the community replication; TEPS-Ant = Temporal Experience of Pleasure Scale, Anticipatory; TEPS-Con = TEPS Consummatory; SHAPS = Snaith-Hamilton Pleasure Scale; SAS = Social Anhedonia Scale; PA = Positive Affect subscale from the PANAS (Positive and Negative Affect Scales).

* *p* < .01.

When shared variance with other BDI items was controlled, the pattern of findings differed somewhat. Both BDI-Anh3 and BDI-Anh4 remained statistically significantly correlated, albeit modestly, with other measures of anhedonia and positive affect for the large undergraduate derivation sample (Sample A), but this was less true for the smaller community replication sample (Sample B). Indeed, only BDI-Anh3 was modestly related to social anhedonia after controlling for the shared variance with other BDI items for Sample B. That the BDI-derived anhedonia scales were more strongly related to the remaining BDI items for Sample B may have contributed to this pattern of findings. That is, the partial correlations removed a greater part of the variance in BDI-Anh3 and BDI-Anh4 for the community sample. As in the analyses of the zero-order correlations, the signs of all the partial correlations were in the expected direction except for one that was essentially zero (.01).

A small subset (*n* = 166) of participants in Sample C, the clinical replication sample, completed the PANAS. Here, we found, as predicted, that both BDI-Anh3 and BDI-Anh4 were moderately and strongly related to PA (*r* values of −.40 and −.50, respectively; *p* < .001). After controlling for shared variance with other BDI items, BDI-Anh3 (partial *r* = −.16, *p* = .043) and BDI-Anh4 (partial *r* = −.29, *p* < .001) remained modestly but significantly correlated with PA. In sum, both BDI-derived anhedonia measures exhibit good convergent validity, although this was weaker for the smaller community replication sample when the shared variance between the anhedonia and other items on the BDI was accounted for.

### Discriminant Validity

To test our hypothesis that both BDI-Anh3 and BDI-Anh4 would demonstrate discriminant validity, we computed correlations and partial correlations between the BDI-derived anhedonia scales and measures we used to assess discriminant validity, as shown in Table 5. When zero-order correlations were examined, both BDI-Anh3 and BDI-Anh4 were moderately or strongly related to self-esteem as assessed by the RSES, anxiety (DASS21 Anxiety, BAI), stress (DASS21 Stress), and negative affect (NA) for both the derivation and community replication samples. Importantly, however, the partial correlation findings show that, with the exception of self-esteem, both BDI-Anh3 and BDI-Anh4 were not related to dissimilar constructs after controlling for the shared variance between the anhedonia and other items on the BDI. In other words, although the BDI-derived anhedonia scales were related to self-esteem, anxiety, stress, and negative affect when zero-order calculations were examined (left columns of Table 5), these relationships were no longer statistically significant after controlling for shared variance with the other BDI items. This was also true for the small subset of participants in Sample C (*n* = 166) who completed the PANAS. That is, both BDI-Anh3 and BDI-Anh4 were moderately related to NA in Sample C (*r* values of .44 and .48, respectively; *p* < .001), but this was no longer the case after controlling for the shared variance with other BDI items (partial correlations of .06 and .00, respectively). Anhedonia as assessed by the BDI, then, is not strongly related to self-esteem, anxiety, stress, and negative affect once the overlap with other depression items is removed.

**Table 5:** Discriminant Validity of the BDI-Anh3 and BDI-Anh4.

	Zero-order correlations				Partial correlations			
	BDI-Anh3		BDI-Anh4		BDI-Anh3		BDI-Anh4	
Measures	A	B	A	B	A	B	A	B
RSES	−.55*	−.48*	−.62*	−.54*	−.16*	.17	−.18*	.21*
DASS21 anxiety	.38*	.50*	.45*	.61*	.02	−.04	.04	.10
DASS21 stress	.46*	.53*	.54*	.60*	.05	.05	.09	.10
BAI	.36*	.52*	.43*	.63*	.04	−.06	.06	.07
NA	.31*	.49*	.37*	.57*	−.04	−.02	−.03	.05

*Note.* Partial correlations are between each BDI-derived anhedonia scale and other measures, controlling for the other items on the BDI. BDI = Beck Depression Inventory; BDI-Anh3 = 3-item BDI anhedonia scale; BDI-Anh4 = 4-item BDI anhedonia scale. A = Sample A (*n* = 455), the derivation sample of undergraduates; B = Sample B (*n* = 185), the community replication; RSES = Rosenberg Self-Esteem Scale; DASS21 = Depression Anxiety and Stress Scales, BAI = Beck Anxiety Inventory; PANAS NA = Positive and Negative Affect Scales, Negative Affect.

* *p* < .01.

However, self-esteem remained modestly correlated with the BDI-derived anhedonia scales for both Samples A and B even after controlling for shared variance, suggesting that diminished pleasure may well be linked to lowered self-esteem. In short, with the exception of self-esteem, these differential associations provide strong evidence for the discriminant validity of the BDI-derived anhedonia scales.

## Discussion

We evaluated the psychometric properties of 3- and 4-item anhedonia measures derived from the BDI that are widely used in studies of anhedonia. We found that the internal consistency of both measures was adequate, though better for the 4-item measure. Indeed, scales with more items tend to have higher internal consistency. We also found strong support for the convergent and discriminant validity of the two scales, though the findings varied somewhat across samples.

In a large undergraduate and smaller community sample, we found that both BDI-derived anhedonia measures—the 3-item and 4-item versions—were, as we predicted, related to other anhedonia measures spanning anticipatory, consummatory, social, and general pleasure. In addition, both BDI-derived measures were (negatively) related to positive affect in these samples as well as in a large clinical sample. These findings are comparable to those of other studies that report modest correlations between BDI-Anh3 and the TEPS (Loas et al., 2018) and the SHAPS (Leventhal et al., 2006; Treadway et al., 2009). Together, these findings provide support for the convergent validity of the BDI-derived anhedonia measures.

We found that the 3-item and 4-item measures were strongly correlated with the remaining BDI items, sharing between 41% and 69% of the variance with those items, a finding comparable to that reported by Leventhal et al. (2006), who found that BDI-Anh3 shared 52% of the variance with other BDI items. Given this overlap, we examined the linkages between BDI-anhedonia and the other similar measures after controlling for this shared variance among BDI items, something that other studies have not done. We found that partial correlations between both BDI-Anh3 and BDI-Anh4 and other measures of anhedonia and positive affect were reduced but remained statistically significant, particularly for the large undergraduate sample. It is unclear why the BDI-derived scales shared more variance with the other BDI items for the AMT community sample compared with the undergraduate or clinical samples. That is, we cannot ascertain whether this may be true for other community samples or may be specific to AMT participants. It would be useful to examine these relationships in other, larger community samples to answer this question. Descriptively, the means, standard deviations, and alphas for the BDI in our AMT sample are comparable to other AMT samples (e.g., Shapiro et al., 2013).

We also found evidence for the BDI-derived measures’ discriminant validity, showing that after controlling for shared variance with other BDI items, neither BDI-Anh3 nor BDI-Anh4 were related to measures of stress, anxiety, or negative affect. Few other studies have assessed correlations between BDI-derived anhedonia and negative affect measures. Leventhal et al. (2006) reported a correlation of .39 between BDI-Anh3 and the Beck Anxiety Inventory, a finding similar to ours (see Table 5). Treadway et al. (2009) reported a correlation of .16 between BDI-Anh3 and NA in a college student sample. Neither of these studies included partial correlations or regression analyses to demonstrate the unique relationship between BDI-derived anhedonia and negative affect, a feature of the present study.

As with any study, it is important to acknowledge key limitations. First, we examined anhedonia scales derived from the BDI and not the BDI-II. Given that the BDI-II is the most recent revision of the BDI and is used in many research studies and clinical settings, it would be useful to assess the psychometric properties of BDI-Anh3 and BDI-Anh4 with BDI-II. Indeed, some studies have already used the 3- and 4-item scales derived from BDI-II. Given the strong correspondence between items on BDI and BDI-II (Beck, Steer, Ball, & Ranieri, 1996), we would anticipate that the results are likely to be comparable. Indeed, Beck et al. (1996) reported pairs of correlations between items from the BDI and BDI-II, and these ranged from .60 to .80 for the anhedonia items. The wording of anhedonia items in the BDI and BDI-II differs slightly except that Item 21 (interest in sex) is identical in the two versions. Item 4 in the BDI assesses satisfaction, whereas in BDI-II it assesses pleasure. Item 12 in the BDI assesses loss of interest in other people, and in BDI-II it assesses loss of interest in other people and activities. Item 15 is the most changed in terms of wording, with BDI assessing effort and BDI-II assessing loss of energy. Thus, it may be particularly useful to examine the 4-item BDI-derived scale using the BDI-II. Because we did not include Item 9 (suicidality) in our derivation and replication samples, it will be useful for future studies to examine the relationship between the BDI-derived anhedonia scales and this item given that this has been a topic of interest in other investigations (e.g., Loas et al., 2018; Winer et al., 2014). Nevertheless, one item from the BDI is not an adequate assessment of suicidality as it does not fully capture intent, means, ideation, or prior attempts, and several other investigations of anhedonia and suicide have used more comprehensive measures of suicide (see meta-analysis by Ducasse et al., 2018). Finally, we did not report factor analytic results. This is largely due to the mixed and messy literature on the factor structure of the BDI (e.g., Fried et al., 2022). In perhaps the most definitive review of BDI factor analyses, Ward (2006) argued that the best-fitting solution is a general factor, *G*, and that the BDI factor structure found in different studies depends largely on sample characteristics (e.g., clinical versus community sample).

Several of our findings differed between the derivation and community replication samples. Our derivation sample was a large college student sample; the community replication sample was smaller and was from AMT. It is difficult to ascertain whether the divergent findings reflect differences in sample size, the sample compositions, or both. To strengthen the quality of data we received from the AMT sample, we followed what could be generally referred to as best practices with the AMT sample. That is, we only recruited those who had completed over 50 prior tasks with a 95% acceptance rate. Indeed, a key metric in the AMT community is reputation, a function of the percentage of a worker’s output that has been approved by requesters. Because a requester can unilaterally reject a worker’s performance on a given task, both immediately canceling the worker’s compensation and lowering the worker’s reputation, there is a strong incentive for workers to complete tasks carefully (Peer et al., 2014). In fact, AMT has advantages relative to other online recruitment, including more stringent identity-verification measures and a strong community norm of high-quality work (Buhrmester et al., 2018). We took further precautions with our sample, as is done in other studies using AMT samples, by excluding those who originated from a suspicious ISP or from duplicate IP addresses. This lowered our sample size but bolstered confidence in the samples’ responses. Nevertheless, it would be worthwhile to do additional work with larger community samples.

Although we were able to include a large clinical sample to evaluate some aspects of BDI-Anh3 and BDI-Anh4, we were not able to fully evaluate the convergent and discriminant validity with this sample. Thus, additional assessment of the validity of these measures with clinical samples would be valuable. Moreover, it would be useful to examine whether the BDI-Anh scales are useful predictors of clinical outcome. Other measures of anhedonia, narrowly or broadly construed, have been potent predictors of outcomes, particularly for depression (e.g., Khazanov et al., 2020; McMakin et al., 2012; Uher et al., 2012). It will also be useful to examine linkages between the BDI-Anh scales—derived exclusively from one self-report measure—and the “interest and activity” facet derived from factor analytic work using the BDI and two-clinician rated measures in the Genome-Based Therapeutic Drugs for Depression (GENDEP) study (Uher et al., 2008). While Uher and colleagues’“interest and activity” includes all four BDI-Anh items, it also includes items less central to or even distinct from anhedonia (e.g., decision-making, tiredness, inability to feel; see Uher et al., 2008, 2012).

In summary, both BDI-derived anhedonia scales are psychometrically sound enough to use in research and clinical settings. Given that the internal consistency is higher for the 4-item version and that it does not add additional burden to complete one more item, we recommend use of BDI-Anh4 over BDI-Anh3, recognizing that a validation study of the BDI-II would be useful to confirm this recommendation with that measure as well. Given evidence that anhedonia is an important treatment target and outcome (e.g., Khazanov et al., 2020), BDI-Anh4 may prove useful as a weekly monitoring assessment tool. Indeed, in clinical settings, assessing anhedonia using the BDI-Anh4 may be particularly useful and efficient if the BDI is already being administered. By contrast, other anhedonia measures contain more than four items. Of course, measures designed at the outset to assess anhedonia likely provide a more comprehensive assessment of anhedonia than BDI-Anh4. Thus, depending upon the research question or clinical context, such a measure may well be more applicable. Nevertheless, for those wishing to assess anhedonia, whether with archival data, in a longitudinal treatment study, or in other research or clinical scenarios, the BDI-derived measures of anhedonia can be solid choices.

## Notes

1. A few studies have used a 2-item scale (Gabbay et al., 2013; Gabbay, Ely, et al., 2012; Gabbay, Mao, et al., 2012; Henderson et al., 2013; Loas et al., 2009; Yaseen et al., 2016), a 5-item scale (Dunn et al., 2009), or do not report the number of items in the BDI-derived anhedonia scale used (Dekeuleneer et al., 2021; Satterthwaite et al., 2015). Two studies have used a different 3-item scale that includes Items 4, 12, and 15, and not Item 21 (Loas et al., 2018; Nizet et al., 2018).

2. The BDI was first published in 1961 (Beck, 1961), and an amended version was copyrighted in 1978 (referred to by Beck et al. as BDI-IA; Beck et al., 1979). The BDI-II was released in 1996 (Beck, Steer, & Brown, 1996). Differences between the 1978 BDI and the BDI-II are relatively small: Three items were replaced, and other items had minor wording changes. Beck, Steer, Ball, and Ranieri (1996) compared the two versions and found each item was strongly correlated with its counterpart (see Beck, Steer, Ball, & Ranieri, 1996, Table 1). Both the 1978 BDI and BDI-II remain in use. The 1978 BDI is most always what is used in studies referring to the BDI (although it is frequently mis-cited with the 1961 reference). When Joiner and colleagues proposed a three-item BDI-Anh measure in 2003, they used the 1978 BDI. In our review of the literature, we found that investigators do not always report whether they used the 1978 BDI or BDI-II, and most cite the 1961 reference regardless of version used.

3. While we would have preferred to include Item 9, the ensuing requirements set forth by our institutional review board would not have been possible to meet given the anonymity of participants in our student and community samples.
